# Co-production of guidance and resources to implement principled participant information leaflets (PrinciPILs)

**DOI:** 10.3310/nihropenres.13423.1

**Published:** 2023-08-21

**Authors:** Nina Jacob, Jeremy Howick, Martina Svobodova, Shaun Treweek, Katie Gillies, Adrian Edwards, Peter Bower, Jennifer Bostock, Kerenza Hood

**Affiliations:** 1 Centre for Trials Research, Cardiff University, Cardiff, Wales, UK; 2 Stoneygate Centre for Empathic Healthcare,, University of Leicester, Leicester, England, UK; 3 Health Services Research Unit, University of Aberdeen, Aberdeen, Scotland, UK; 4 Division of Population Medicine, Cardiff University, Cardiff, Wales, UK; 5 Division of Population Health, Health Services Research & Primary Care, The University of Manchester, Manchester, England, UK; 6 Lay Research Advisor, Centre for Trials Research, Cardiff University, Cardiff, Wales, UK

**Keywords:** Co-production, patient involvement, harms, nocebo, placebo, research ethics, adverse events, participant information leaflet

## Abstract

**Background:**

The way information about potential benefits and harms of trial is presented within participant information leaflets (PILs) varies widely and may cause unnecessary ‘nocebo’ effects. The Medical Research Council (MRC) funded a project that developed seven principles to reduce this variation. However, guidance has not been produced to facilitate the implementation of the principles. Stakeholder involvement is recommended to optimise the way these principles are disseminated and explained. To co-produce recommendations for developing: (1) user-friendly guidance for users of the principles; and (2) resources that support the implementation of the principles.

**Methods:**

We held a co-production workshop with representation from the following professional groups: the Health Research Authority (HRA), research ethics committee members, and trial managers. Two rounds of discussions focused on generating recommendations for guidance and resources that support the implementation of the seven principles. Extensive low inference style ethnographic notes were taken, and the data were analysed thematically using deductive codes. The data was collected on October 14, 2022.

**Results:**

25 participants attended a hybrid workshop. Participants recommended that both researchers designing PILs and research ethics committee members should use the principles, and that that they should be simple, mention both benefits and harms explicitly, include examples of visual representations, and provide the evidence base for the principles.

**Conclusions:**

We were able to co-produce recommendations for developing and implementing the seven principles within PILs. These recommendations can now be implemented to reduce unexplained variation in the way potential benefits and harms are shared within PILs.

## Plain language summary

Trial participants need to know about the potential benefits and harms of trial interventions to make an informed decision about whether to take part in a clinical trial. Yet the way they are told about these benefits and harms varies widely. We developed seven principles that can reduced this variability. We held a workshop with members of ethics committees and other stakeholders to develop the best way to implement the principles. 25 participants attended the workshop, and we were able to make useful recommendations that will improve the way trial participants are told about risks and benefits of trial treatments.

## Introduction

Trial participants need to know about the potential benefits and harms of trial interventions to make an informed decision about whether to take part in a clinical trial ^1, 2^. However, research has shown that the way such information is shared within participant information leaflets (PILs) varies widely and is often unbalanced, with potential treatment benefits frequently not mentioned at all ^3^. Additionally, overemphasising harms can induce unnecessary information-induced harm (‘nocebo’ effects) ^4^, which in turn may adversely affect trial recruitment ^5^.

To help reduce this variation and unnecessary nocebo harms, the Medical Research Council funded a project called ‘Developing and Testing Participant Information Leaflets that Inform and Do Not Cause Harm (PrinciPIL)’ ^6, 7^. This project included a Delphi process involving a range of stakeholders (participant representatives, ethics committee members, industry representatives, medico-legal experts, applied researchers, research nurses and trial managers) to identify principles that can be used to guide the way in which information about potential benefits and harms of trial interventions is shared within PILs. The principles are as follows:

1. All potential harms of the intervention should be listed.
2. The harms should be separated into serious (life threatening, causing permanent damage) and less serious (like a mild headache that goes away quickly).
3. The fact that not all potential harms are known needs to be explicit.
4. All potential benefits of the intervention should be listed.
5. The potential benefits and harms of a clinical trial need to be compared with what happens if the participant does not take part in the trial.
6. Suitable visual representations are recommended where appropriate to describe potential intervention benefits and harms, such as pictograms of faces.
7. Information about potential benefits and harms should be presented in proximity (for example, on the same page).

PILs that are informed by the principles above are called PrinciPILs, and PrinciPILs are designed to have three main benefits. First, they will reduce the variability in the way information about the potential benefits and harms of trial treatments is described within PILs ^3^. Secondly—and this is a consequence of the above—the principles have the potential to reduce research waste arising from different trial and research ethics committee (institutional review board) teams, developing their own ‘best’ way to share information about the potential benefits and harms of trial treatments. Third, they may improve recruitment rates and reduce nocebo effect harms. To test this, PrinciPILs are being compared with standard PILs in a series of studies within a trial (SWATs) to evaluate whether they influence recruitment rates and trial-related clinical outcomes (especially participant-reported harms, which may be nocebo effects) ^8^.

Given their likely benefits, it is important to implement the principles with user-friendly guidance that researchers who design PILs and ethics committees who evaluate them understand and can use. When developing guidance, stakeholder engagement is considered crucial for ensuring priority issues are considered and identified ^9^. The importance of involving practitioners and other stakeholders is also considered key to ensuring guidance is adopted, implemented and maintained in the contexts for which it is intended ^10, 11^.

### Aims

To co-produce recommendations for developing:

1. user-friendly guidance for users of the principles; and
2. resources that support the implementation of the principles.

## Methods

### Ethical statement

In accordance with Health Research Authority (HRA) guidance, this study did not require ethical approval as this study was conducted with professionals to discuss their professional opinions. Attendees (all professionals) provided their verbal consent to publish the results of the workshop at the outset of the workshop. The sponsor of the study agreed that oral consent sufficed, given that no patients were involved in the workshop, no patients data was included, and because the workshop attendees were all acting in their professional capacities and providing their views related to their professional work.

### Patient and public involvement

A patient and public involvement (PPI) representative (JB) was involved in acquiring the funding for this study, question development, research design, and background research. The same PPI representative is involved in our ongoing active dissemination plan for this study.

### Recruitment of the co-production group

Participants were chosen from the group of stakeholders (patient and public representatives, research ethics committee members, industry representatives, medico-legal experts, psychologists, and trial managers) who had registered their interest in designing, evaluating, or using PILs from a previous study ^7^. We then used a purposeful sampling strategy to select participants, and contacted them via email. A purposeful strategy helped ensure that group members were drawn from a range of networks whilst also ensuring representation of stakeholders from key organisations who examine PILs (HRA and research ethics committee members) and those who design them (trial managers and other clinical trial representatives). A choice of attending virtually or in person was offered to encourage maximum participation, although most stakeholders attended in person.

### Co-production workshop

In advance of the face-to-face workshop held on October 14 ^th^, 2022, an email was sent to specify that the scope of the workshop was to discuss the guidance required alongside the principles and suggestions for required implementation resources. It was also specified that a discussion of the principles themselves was beyond the scope of the workshop, as they had already been developed with extensive stakeholder input ^12^.

Verbal consent to publish the results was obtained at the outset of the workshop. We followed the methodology used in a related project ^13^ The research team presented an overview of the evidence underpinning the principles (JH). A brief general discussion of the PrinciPIL findings followed. Next, small groups (n=3) were chosen at random to discuss the two questions listed below.

1. How can we best present the principles so that they can be easily understood and implemented?
2. What resources (for example, web-based resources) are required to support implementation of the principles?

Iterative rounds of feedback and discussion were conducted after each question, with opportunities to raise conflicting opinions provided. One researcher (NJ) was tasked to take detailed notes during the day. This included minuting of the reports from the small groups. Key points were reflected and summarised, and areas of widespread agreement and of disagreement were noted.

### Analysis

As part of the co-production workshop, extensive low inference style ethnographic notes were taken and then expanded upon following the meeting. Data were analysed thematically using deductive codes that had been identified prior to the workshop, based on the two main questions asked during the workshop. Additional inductive codes relating more broadly to the seven core principles were added where required ^14^. For the purpose of this study, the analysis concentrated on: (i) feedback on the two main questions asked of the group; (ii) the stakeholders’ broad observations on the utility of the seven core principles; and (iii) how the feedback can be incorporated into the main principles.

## Results

### Demographics of the co-production group

We were able to meet our target number of attendees (n=25). The attendees came from the East Midlands, Southeast, London, Yorkshire and the Humber, and the Southwest. There were 19 female attendees (76%), and five (20%) were from non-White backgrounds.

### Presenting principles

In discussing how best to present the principles so that they can be easily understood and implemented, five main themes arose. These focused on the need for (i) the guidance to be easily understood, (ii) clarification of the proper use of appendices, (iii) examples of appropriate visual representation, (iv) provision of the rationale for describing potential benefits, and (v) the need to highlight the evidence base underpinning the principles.

***Guidance should be useable by all researchers designing PILs ethics committee members.*** Whilst some welcomed a single guidance document understandable for both applicants and reviewers, others questioned whether a one-size-fits-all document would be appropriate. For research teams, this could come in the form of a standard operating procedure (SOP) or similar protocol. For research ethics committees, it was recommended that the PrinciPIL guidance should serve as a template for a conversation with research teams. The usual practice for research ethics committees is to accept the risks and benefits presented to them by study teams. It was suggested that the PrinciPIL guidance could thus serve as a change in practice by encouraging a conversation around what has been included and why. It was also suggested that a different approach might be needed for different trials. It was noted that the guidance had to take into consideration that PILs for different trials and trial populations needed to be different. For example, explaining risks and benefits to children is likely to be very different from explaining risks and benefits to adults.

***The proper use of appendices needs to be clarified.*** Appendices are sometimes used to avoid overly lengthy risks and benefits sections in certain trials. The extent to which these are accessed by participants was queried, although it was agreed that their use may sometimes be required to ensure readability.

***Examples of visual representation should be provided.*** The types of visual representation of risks that would be appropriate were not clear to the attendees. The attendees recommended that examples of visual representation be provided. One participant queried the use of smiley faces and suggested using pictograms that reference the text as an alternative approach. 

***The rationale for including information about potential benefits should be clear.*** A great deal of discussion centred on whether the potential benefits of trial treatments should be mentioned at all within PILs. Some of the participants claimed that potential benefits are subjective and not known (whereas many harms were considered to be known). In addition, the group thought it would be useful to think about benefits as direct (from the intervention i.e., therapeutic) and indirect (wider participation in research i.e., non-therapeutic) and to be clear about what the benefits listed are.

***The evidence base underpinning the principles must be highlighted.*** Many workshop attendees had not done the suggested background reading and were unaware of the evidence underpinning the principles. Stakeholders therefore considered it useful to be reminded of the evidence base that underpins the seven principles ^3, 4, 12^.

### Specific feedback relating to the core principles

The group was invited to make specific suggestions with regards to the guidance for the principles. Below is a summary of the main points of discussion and will be incorporated into the PrinciPIL guidance (see Table 1).

**Table 1.** Proposed actions based on results of co-production workshop.

Category	Suggestion	How we are taking suggestion into account in the development of dissemination of the principles
**Presenting** **principles**	Guidance should be usable by all ethics committee members and researchers designing PILs	On our website we make it clear that the principles are to be used as a helpful guide and that the way and extent to which they are implemented will vary from trial to trial depending on several factors.
	The proper use of appendices needs to be clarified	We have taken this into account by making it clear in the PrinciPILs that (a) not all potential harms are known, and (where applicable) that (b) a complete list of known potential harms is contained in an appendix and (c) that additional information can be obtained from trial team.
	Examples of visual representation should be provided	The way we present potential benefits and potential risks includes visual elements such as contrasting colour. On our website, we have also listed the types of visual representations and provided references.
	The rationale for including information about potential benefits should be clear	We have written a paper citing regulations from the UK, US, and EU where it is clearly stated that mentioning potential benefits of a trial intervention (where they exist) is required ^15– 17^.
	The evidence-base underpinning the principles must be highlighted	On our website we have included a description of our extensive background studies.
**Specific feedback** **on principles**	The source of potential risks should be made clear	We have included this in our guidance document.
	An individualised approach needs to be adopted when separating the harms into serious and less serious	On our website we make it clear that the principles are to be used as a helpful guide and that the way and extent to which they are implemented will vary from trial to trial depending on several factors.
	The sources of potential benefits need to be made explicit.	On our website, we have added an explanation of where information about potential benefits is likely to be found.
	The answer to the question of what the risks of not taking part in the study requires elaboration.	We recommend that, where applicable, the following standard statement be added to the PrinciPILs: ‘if you do not come into this trial, you will get standard care. To learn more about the risks and benefits of standard care please ask the trial team or your physician.’
**Resources for** **implementing** **principles**	Using resources, training, webinar, and a SOP amendment for CTUs	We have developed several resources including a website, a video, and recommendations for amending SOPs. For all of these, take measures to discourage slavish adherence to principles that does not account for the need for adaptation to specific trials and trial populations.
	Keep it simple	We developed infographics for the principles that are the key features of our website.

Abbreviations: SOP=standard operating procedure; CTU=clinical trials unit

**1.**  **All potential harms should be listed**: The group agreed with the importance of this principle, especially because ethics committee members are seldom qualified in the disciplines they review. For similar reasons, it was suggested that accompanying guidance should include information on appropriate sources of information for non-drug studies. For example, Cancer Research UK (CRUK) has specific support groups to ensure that a variety of different pathways of information are sourced. It was suggested that an appendix or link should be used for risks over a specified amount.
**2.**  **The harms should be separated into serious (life threatening, causing permanent damage) and less serious (like a mild headache that goes away quickly)**: Participants agreed with this principle and noted that an individualised approach needs to be taken during additional consent conversations.
**3.**  **The fact that not all potential harms are known needs to be clear**: No suggestions were made regarding guidance related to this principle.
**4.**  **All potential benefits of the intervention should be listed**: It was suggested that clear guidance was required to define what constitutes a benefit and where the evidence for the benefit came from. In addition, participants suggested that direct potential benefits (that might arise from the intervention) be delineated from indirect benefits (wider participation in research).
**5.**  **Potential benefits and harms of a clinical trial need to be compared with what happens if the participant does not take part in the trial**: A suggestion was made to present this information such as ‘Consequences of taking part and the accompanying risks and benefits.’
**6.**  **Suitable visual representations are recommended where appropriate to describe potential intervention benefits and harms, such as pictograms of faces**: No additional suggestions were made regarding this principle. It was reiterated that examples would be useful.
**7.**  **Information about potential benefits and harms should not be presented apart by one or more pages**: There was unanimous agreement that this was a good idea, and there were no suggestions for guidance related to this principle.

### Resources for implementing the principles

When discussing how to support the implementation of the principles, several suggestions were made, including the use of resources such as webinars and SOPs. It was also recommended that we keep things simple, with one participant stating that a simple list of the seven principles may suffice. We have described these below.

***Use of resources/training/webinars/SOPs for clinical trials units.*** Participants noted that appropriate implementation of resources is relative to the target audience. For example, researchers need more detailed instructions, whereas ethics committees may require exemplars. When developing resources, it would be useful to test these out with different research ethics committees. Several attendees noted that the implementation resources should not encourage slavish and unthinking adherence to principles. It was suggested that this could be achieved with worked examples.

***Keep it simple.*** Participants agreed that a strength of the principles is their brevity. It was even suggested that a single-page explanation of the seven principles could suffice.

### Implementing the suggestions into the PrinciPIL project

We implemented all the suggestions made at the workshop to improve the way the principles are disseminated and explained (see Table 1).

### Wider issues

Whilst the focus of the workshop was guidance and implementation, several wider points were raised during the workshop. We agreed to make a note of these for future research, and they are described below:

**1.**  **Importance of the broader consent procedure**: The PrinciPIL approach is focussed upon the written communication around consent practices. It was noted that oral communications surrounding consent are at least as important ^18^. Relatedly, the trial participants’ understanding of likelihood and risk was likely to be variable, and an individualised approach needs to be taken during consent conversations.
**2.**  **Are all harms and benefits equal?** It was thought that it would be beneficial to think beyond the potential benefits and harms of the intervention itself to more general considerations, including time taken, increased monitoring and jumping queues. The potential to consider benefits to organisations (such as research organisations that generate income from research and pharmacological companies) was also raised, although no consensus was achieved on this point.
**3.**  **The legality of mentioning potential benefits**: A vocal minority of participants stated that European guidance claimed to recommend against mentioning potential trial treatment benefits within PILs. This was disputed in the workshop and was subsequently found to be false. The European Union clinical trials regulations state that mentioning benefits is required:
  ‘Information given to the subject or, where the subject is not able to give informed consent, his or her legally designated representative for the purposes of obtaining his or her informed consent shall: (a) enable the subject or his or her legally designated representative to understand: (i) the nature, objectives, benefits, implications, risks and inconveniences of the clinical trial.’ ^15^
**3.**  **Greater definition about what constitutes a risk:**The way risks (of trial treatments) are defined varies widely, and there is no consensus around the best way to define or present them ^19^. There was general agreement that natural frequencies should be used (whole numbers rather than percentages), and this needs to be made clear in the guidance.
**4.**  **Rationale for taking part in the trial**. Some of the attendees considered it essential for the PIL to answer to the question ‘Why should I take part in this trial and what will happen if I don’t?’ at the beginning of the PIL. As this goes beyond the scope of our current project, we did not incorporate it into our plans and instead made note of it for future research.

## Discussion

### Summary of results and general interpretation

We were able to co-produce several recommendations for PIL guidance and resources to support the implementation of the seven principles that can harmonise the way in which information about potential intervention benefits and harms is shared within PILs. The suggestions included the following: ensuring that guidance was useable by both research ethics committees and researchers; highlighting the evidence base underpinning the principles; the need for clarity regarding the use of appendices; the need for clarity regarding appropriate visual representation; and the need for greater clarity regarding the discussion of potential benefits. Overall, the group considered the simplicity of the principles to be their strength and, as a result, that minimal additional resources were required to implement them.

### Comparison with related research

The need to reduce variability in the application of research ethics principles has been recognized ^20^, and calls have been made to harmonize research ethics guidance ^6, 21^. Unfortunately, and with few exceptions ^22^, guidance for sharing potential trial intervention benefits and harms is under-researched ^7^. By providing suggestions that can reduce the variability in the way in which potential trial intervention benefits and harms can be shared within PILs, our study addresses the identified need to harmonize research ethics guidance.

### Strengths and weaknesses of the study

A limitation of our co-production workshop was that the attendees had not done the required background reading outlining evidence that underpinned the seven principles. This led to tangential discussions, especially about the need to mention potential benefits, which threatened to undermine the groups’ confidence in the value of the principles.

Future research should evaluate the extent to which the guidance and resources we produce are used and able to reduce the variability in the way potential benefits and harms are described within PILs. In addition, our methodology can now be expanded. Whereas our principles were applicable to written materials, we can now develop parallel principles that can inform verbal discussions of the potential benefits and harms of trial treatments. Additionally, there is a need to overcome the mistaken belief that mentioning potential trial/intervention benefits within PILs is unlawful.

## Conclusion

Stakeholders were able to co-produce suggestions for guidance and resources for implementing the seven principles that will harmonise the way information about potential trial intervention benefits and harms are described within PILs. These principles have the potential to help trial teams and research ethics committees consider what risks and benefits should be included, reduce variation in the way these are communicated and reduce research waste arising from both trial teams and research ethics committees having to develop their own optimal way to share this information. The recommendations can now be implemented.

## Data Availability

The workshop records used in this study are restricted to ensure the anonymity of the participants due to the small sample size. Ethical approval and consent was obtained on the basis that data would remain anonymous. Preserving anonymity requires that additional data not be shared. All unrestricted data is available in the manuscript, any additional data sharing would de-anonymise the sample. To request access to the restricted data, please contact Professor Jeremy Howick ( jh815@leicester.ac.uk).
