# Supplementary material for: Co-production of guidance and resources to implement principled participant information leaflets (PrinciPILs)
Source: NIHR Open Res. 2023 Aug 21;3:42. [Version 1] doi: 10.3310/nihropenres.13423.1 (PMC11406117; doi:10.3310/nihropenres.13423.1)
Supplement: Peer review history

# Reviewer report: Reviewer response for version 1

**DOI:** 10.3310/nihropenres.14557.r30610

**Published:** 2024-09-16

**Authors:** Gonasagrie (Lulu) Nair

**Affiliations:** 1 Desmond Tutu Health Foundation, Mowbray, South Africa

This is a very useful study and will be of relevance to those who develop PILs and for ethics committees who review them. 

 The work is clearly presented. However, the literature cited appears to be restricted to the developed world context. More and more frequently clinical trials are now multisite, spanning both developed and developing countries. To make the recommendations generalizable and globally relevant, perhaps views of stakeholders/experts from developing countries could have been included. Cultural beliefs and practices play a major role in adoption of or adherence to interventions under investigation in trials. This is especially. true for prevention studies/trials among healthy volunteers (e.g non adherence to study product among young women in HIV prevention trials in SSA Africa). 

 The co-production workshop is an appropriate design to solicit feedback on the development of guidance and resources for implementing the 7 principles. However, more information is required on the characteristics of the workshop participants: Were community members or community advisory board members included?  Noted also that participants were allowed to join online and it would have been feasible to allow participants from developing countries to join, making the feedback more broadly relevant. 

 The justification of not making source data available to preserve anonymity is acceptable but the researchers could have considered including comments from participants made during the discussions to support the conclusions. For example, what were thoughts on what constitutes a benefit? Should "jumping the queue" or receiving a service at no cost such as contraceptive access or a screening procedure be considered a benefit. Or should a benefit be restricted to that of using/adhering to the experimental drug. 

 Did any of the discussion focus on the access to resources/guidance tools in different settings and how likely busy ethics committee members who are often volunteers would seek out these resources. 

 I agree that the strength is the simplicity of the principles and look forward to seeing the accompanying guidelines and resources.

Is the work clearly and accurately presented and does it cite the current literature?

Yes

If applicable, is the statistical analysis and its interpretation appropriate?

Yes

Are all the source data underlying the results available to ensure full reproducibility?

No source data required

Is the study design appropriate and is the work technically sound?

Yes

Are the conclusions drawn adequately supported by the results?

Yes

Are sufficient details of methods and analysis provided to allow replication by others?

Yes

Reviewer Expertise:

HIV and TB treatment and prevention clinical trials. Research Ethics. I have a keen interest in the informed consent process and am currently a member of a research ethics committee.

I confirm that I have read this submission and believe that I have an appropriate level of expertise to confirm that it is of an acceptable scientific standard.

# Reviewer report: Reviewer response for version 1

**DOI:** 10.3310/nihropenres.14557.r30404

**Published:** 2023-10-25

**Authors:** Stuart Nicholls

**Affiliations:** 1 Ottawa Hospital Research Institute, Ottawa, Canada

**Co-production of guidance and resources to implement principled participant information leaflets (PrinciPILs)**

 In the present manuscript the authors provide an overview of a workshop undertaken to inform the development of guidance documents for the implementation of the PrinciPIL principles. Improving the consent process to ensure potential participants can make informed decisions is a key ethical marker of research and work to improve this process is important. I do, however, have some specific comments for consideration (listed under the section headings) as well as several larger comments for the authors to consider. In terms of the larger comments:

1. The workshop is focused on developing user friendly guidance to improve the use and evaluation of PILs. The Principles themselves are framed in terms of impact on trials: Yet data is not presented as to whether PILs designed in accordance with the PrinciPILs actually achieve the proposed benefits: does the PrinciPIL designed PIL reduce nocebo effects, for example? Should one not have evidence that adherence to the 7 principles does indeed have benefits? Seeking to improve uptake or adherence, absent evidence of benefits (or lack of harms) seems pre-emptive and it is notable that this was not raised as a theme (even though evidence of benefits to be listed in the PIL was).
2. A second aspect I found challenging was that the term ‘user-friendly’ is vague. How does one define or evaluate the ‘user-friendliness’ of the guidance? Was this framed in a specific way for participants? It is relevant as it could relate to language, visuals, format, as well as accessibility. Perhaps the notion of user friendly could be expanded upon or greater specificity in the objectives offered.
3. Finally, can the authors expand more on their definition of co-production? The term is used throughout but the work presented seems more akin to a consultation, unless the individuals engaged are/were also involved in the actions taken (as detailed in the 3rd column of Table 1). If a definition could be provided, along with how the study and future work meets the definition, that would be helpful.

METHODS

 While the paper discusses purposeful sampling, this seems to only reflect on role. Were other  experiential or demographic characteristics used to inform sampling? Was there any representation, for example, from people with design experience or with expertise in knowledge translation/implementation or other skills that could provide input on best approaches to uptake of information?

 ANALYSIS

 What deductive codes were used? How do the final themes relate to the deductive coding vs indictive coding?

 RESULTS

 Given the noted fact that many had not done the background reading on the evidence base for the principles; to what extent can these views be considered informed or deliberated vs initial or reactionary?

 I am also not completely sure on the goal of the analysis, which again goes back to the relatively broad aim. In some parts the results seem to reflect a desire to share the range of issues participants deemed relevant, yet in other areas it is more focused, and in some cases quasi-quantitative in the way that there is reference to “unanimous agreement” or a single suggestion being made.

 Some of the proposals seem somewhat circular to the questions posed. For example, a key question posed was “How can we best present the principles so that they can be easily understood and implemented?” and a theme in response is to make them useable by REC members and researchers. This seems to go without saying doesn’t it? One wouldn’t make them unusable. It seems to me that the more important question is HOW can they be made useable to researchers and how can they be made useable to RECs. Put the other way, it would seem important to identify what might be barriers to their use?

 The theme of having a rationale for including benefits is interesting as it seems to in part be more about refining the principle itself (e.g. the specification as direct and indirect benefits) as opposed to a way to present the principle so it can be understood. This is consistent with the serious/less serious specification of harms and how that is incorporated into the principles themselves.

 In addition, there seems to be some overlap between the theme relating to the rationale for including benefits, and the specific comments about listing the potential benefits of an intervention (both for example, discuss direct and indirect benefits). Perhaps these sets of results could be merged rather than duplicated.

 I am not clear on the concerns about “slavish and unthinking adherence to principles.” Is that not what is exactly desired – that these principles are universally adopted? If not, would that not lead to the variation in content that is lamented in the previous review and the introduction? Indeed, the fact that they are principles and not actions seems to require consideration and thought about how to they are applied in practice. Or was it the case that the comment reflects concern for a focus solely on these principles to the detriment of considering other important information? Perhaps clarifying this or offering perspective would be helpful.

 There is also variation in the results reporting. For some comments the results are provided solely as description: e.g. a topic was raised. In other areas of the results there is commentary made on the issue (e.g. the legality of mentioning potential benefits). Some are highlighted but then explained as not relevant, so I wasn’t sure of the rationale for raising them in the manuscript results (as opposed to reporting them as issues raised and then addressing them in the discussion as to whether they are misinformed).

 DISCUSSION

 The discussion would benefit from greater integration with the literature. While I realise the focus here is on the suggestions around approaches that can be made to improve the uptake of the PrinciPILs principles there has, for example, been lots of work in the area of participant information leaflets and especially in relation to required content (e.g the US regulations stipulate core components: https://www.hhs.gov/ohrp/regulations-and-policy/guidance/faq/informed-consent/index.html ). This is built into regulations, so it would be useful to contrast the regulatory approach to the integration of content, and this principled approach. Similarly, reviewing the literature relating to the topics raised would be helpful – for example, the visual representation of risks.

Is the work clearly and accurately presented and does it cite the current literature?

Partly

If applicable, is the statistical analysis and its interpretation appropriate?

Not applicable

Are all the source data underlying the results available to ensure full reproducibility?

No

Is the study design appropriate and is the work technically sound?

Yes

Are the conclusions drawn adequately supported by the results?

Partly

Are sufficient details of methods and analysis provided to allow replication by others?

Partly

Reviewer Expertise:

Research ethics, patient-oriented research

I confirm that I have read this submission and believe that I have an appropriate level of expertise to confirm that it is of an acceptable scientific standard, however I have significant reservations, as outlined above.

# Reviewer report: Reviewer response for version 1

**DOI:** 10.3310/nihropenres.14557.r30351

**Published:** 2023-09-15

**Authors:** Melanie Fleming, Barbara Robinson

**Affiliations:** 1 University of Oxford, Oxford, England, UK

This qualitative study aimed to generate and collate suggestions for guidelines of use for a set of principles which were created to help standardise the way in which harms and benefits are presented in participant information leaflets (PILs). Stakeholders across different professional research settings were invited to a workshop to discuss recommendations of guidance based on the principles, and these recommendations were thematically presented.

 The paper is well written, and does a good job of highlighting the importance and need of having consistent and accurate guidelines that can be used by research and ethics teams to implement the principles laid out, and benefits from considering the viewpoints of a diverse range of stakeholders.

 I have several minor comments/suggestions for consideration:

1. Due to the workshop building on the previous work in which the principles were generated, the narrative within the introduction was a little confusing in terms of what this study was doing, and what had already been done (e.g. when mentioning the Delphi process, I initially thought that was in relation to the current study, rather than the principle generation). The introduction would benefit from some rewording to clarify what was past and what is the current work. 
2. The introduction also presents the SWATS, although this is useful and relevant to the work, this information would be better placed in the discussion section of the paper as it seems like “next steps” rather than work leading up to this part of the project. 
3. The methods note that there was a “purposeful sampling strategy” employed. It would be useful to include more detail as to which criteria was used for this sampling. It is currently unclear whether patient representatives were included or not, as the “patient and public involvement” subsection implies that they were not involved in this workshop, but the “recruitment of the co-production group” does mention patient and public representatives as part of the group of stakeholders from which participants were chosen for this study.
4. The authors note that many of the workshop attendees did not read the background reading, however there was no description of the background reading provided. Please could details of the suggested reading be included in the methods section.
5. Under the ‘ *wider issues’* section of the results, point 2 mentions benefits/harms beyond the intervention itself. However, this point is somewhat covered in the ‘ *specific feedback relating to core principles*’ section already, and I recommend that this therefore be incorporated into this section (specifically points 2 and 4) rather than being separate.
6. The conclusion points to the guidance being applicable and useable by both researchers creating PILs and ethics review boards. However, given some of the feedback provided in the results (specifically on pg 4: “ *Whilst some welcomed a single guidance document understandable for both applicants and reviewers, others questioned whether a one-size-fits-all document would be appropriate*.”), I am not wholly convinced by this conclusion, and further discussion of this point may be more apt within the paper.

Is the work clearly and accurately presented and does it cite the current literature?

Yes

If applicable, is the statistical analysis and its interpretation appropriate?

Not applicable

Are all the source data underlying the results available to ensure full reproducibility?

Partly

Is the study design appropriate and is the work technically sound?

Yes

Are the conclusions drawn adequately supported by the results?

Yes

Are sufficient details of methods and analysis provided to allow replication by others?

Yes

Reviewer Expertise:

Research involving human participants, including clinical trials. Ethical oversight. Experience in patient and public involvement in research.

We confirm that we have read this submission and believe that we have an appropriate level of expertise to confirm that it is of an acceptable scientific standard, however we have significant reservations, as outlined above.
